# Supplementary material for: Pressure encryption toward physically uncopiable anti-counterfeiting
Source: Nat Commun. 2025 Jul 5;16:6203. doi: 10.1038/s41467-025-61509-3 (PMC12228716; doi:10.1038/s41467-025-61509-3)
Supplement: Supplementary file 1 — Supplementary information [file 41467_2025_61509_MOESM1_ESM.pdf]

Supplementary Information for

Pressure Encryption Toward Physically Uncopiable Anti-Counterfeiting

Dianlong Zhao[1,2,#], Shunxin Li[1,#], Yang Su[3,#], Jiajun Qin[4,#], Guanjun Xiao[1,*], Yuchen Shang[1,5], Xiu Yin[1], Pengfei Lv[1], Feng Wang[1], Jiayi Yang[1], Zhaodong Liu[1,5], Fujun Lan[6], Qiaoshi Zeng[6], Lijun Zhang[3,*], Feng Gao[4,*] and Bo Zou[1,*]

[1]*State Key Laboratory of High Pressure and Superhard Materials, College of Physics, Jilin University, Changchun 130012, China*

[2]*School of Physics and Materials Engineering, Dalian Minzu University, Dalian, 116600, China*

[3]*School College of Materials Science and Engineering, Jilin University, Changchun, 130012, China*

[4]*Department of Physics, Chemistry and Biology (IFM), Linköping University, Linköping 58183, Sweden*

[5]*Synergetic Extreme Condition User Facility Jilin University, Changchun, 130012, China*

[6]*Center for High Pressure Science and Technology Advanced Research, Shanghai 201203, P. R. China; Shanghai Key Laboratory of Material Frontiers Research in Extreme Environments (MFree), Institute for Shanghai Advanced Research in Physical Sciences (SHARPS), Shanghai 201203, P.R. China*

[#] These authors contributed equally to this work.

*Corresponding authors: xguanjun@jlu.edu.cn (G.X.); lijun_zhang@jlu.edu.cn (L.Z.); feng.gao@liu.se (F.G.); zoubo@jlu.edu.cn (B.Z.)

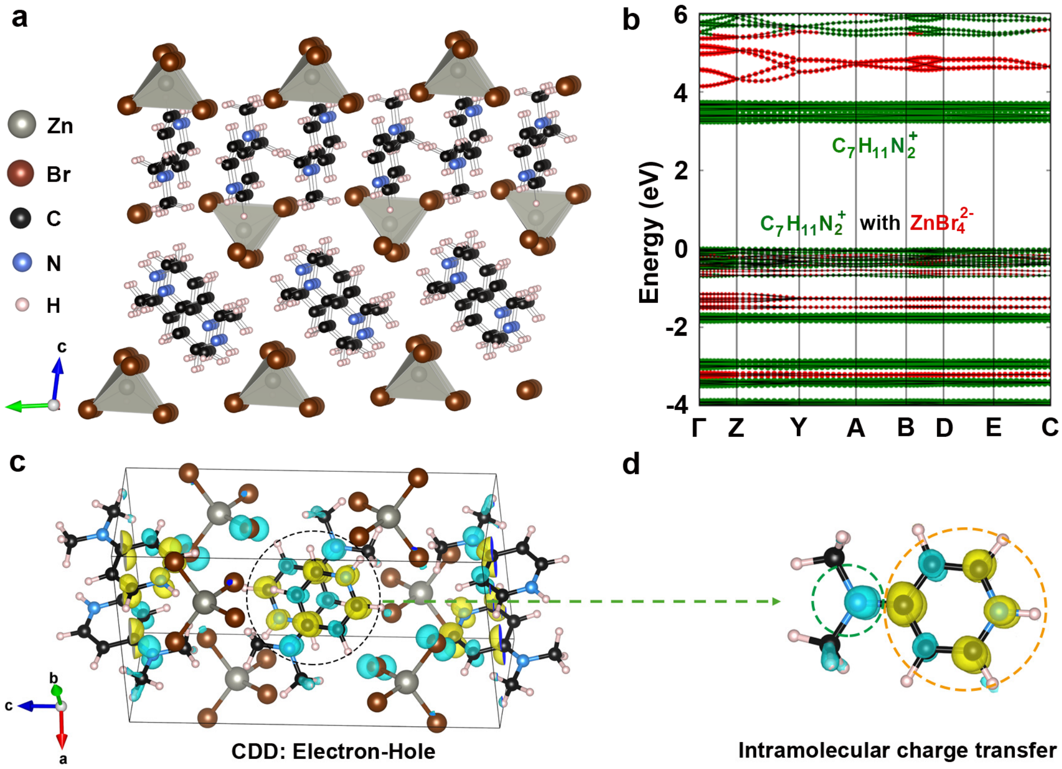

Supplementary Fig. 1 | Characteristics of the crystal structure and electronic properties of $(4DMAP)_2ZnBr_4$ under ambient conditions. **a** Schematic illustration of layer stacking for $(4DMAP)_2ZnBr_4$. **b** Calculated total projection of band structure for $(4DMAP)_2ZnBr_4$. **c** and **d** Calculated difference between electron and hole for $(4DMAP)_2ZnBr_4$, considering the periodicity of the structure.

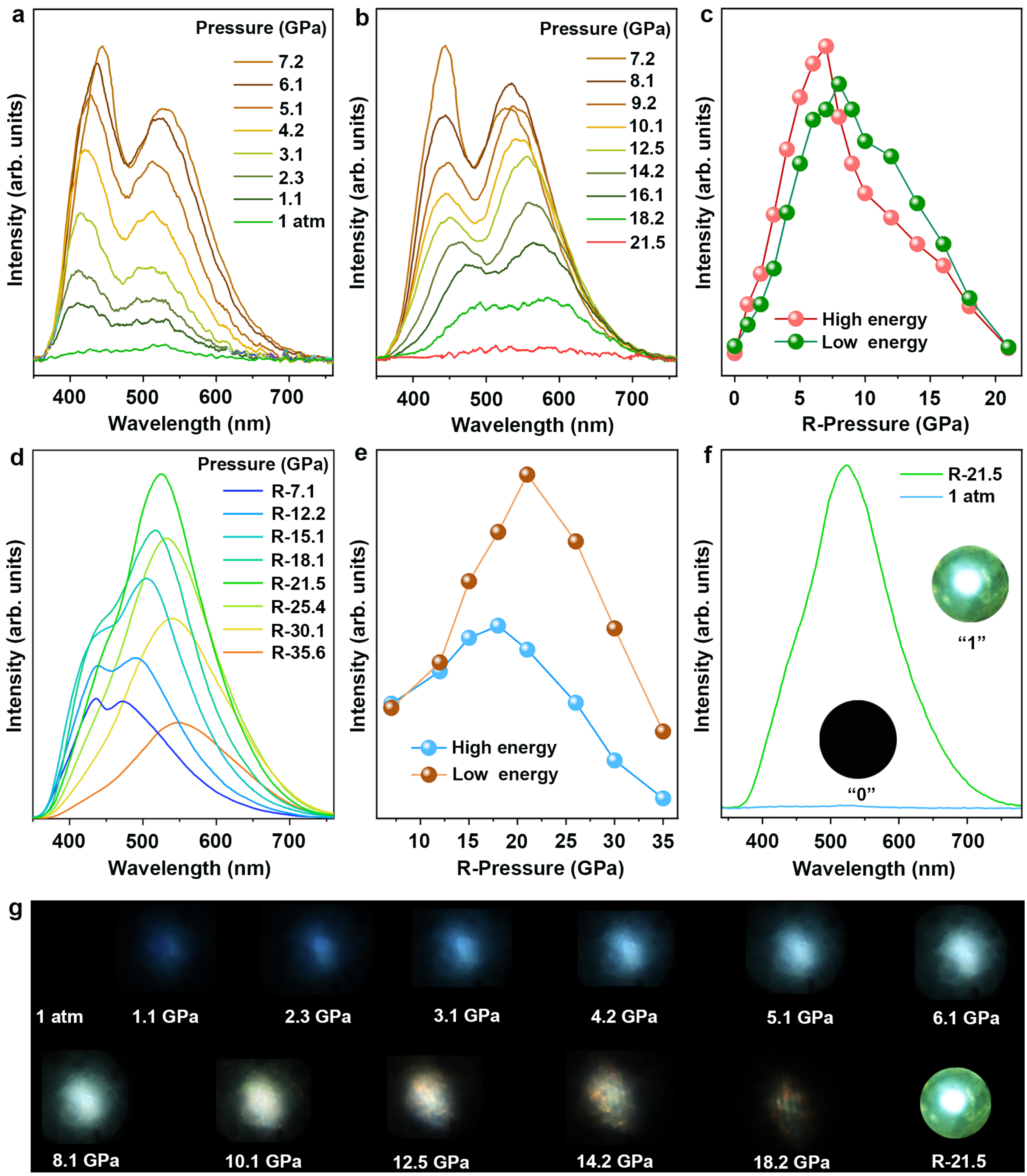

Supplementary Fig. 2 | PIE and Optical photographs. a and **b** Changes in PL spectra of $(4DMAP)_2ZnBr_4$ under high pressure. **c** PL intensity varies in the samples 1 atm, …, 21.5 during loading different pressures. **d** Changes in PL spectra after loading different pressures. **e** PL intensity varies in the samples R-7.1, …, R-35.6 after loading different pressures. **f** Comparison of PL spectra between 1 atm and R-21.5 GPa. **g** Optical image evolution under varying pressures and at R-21.5 GPa.

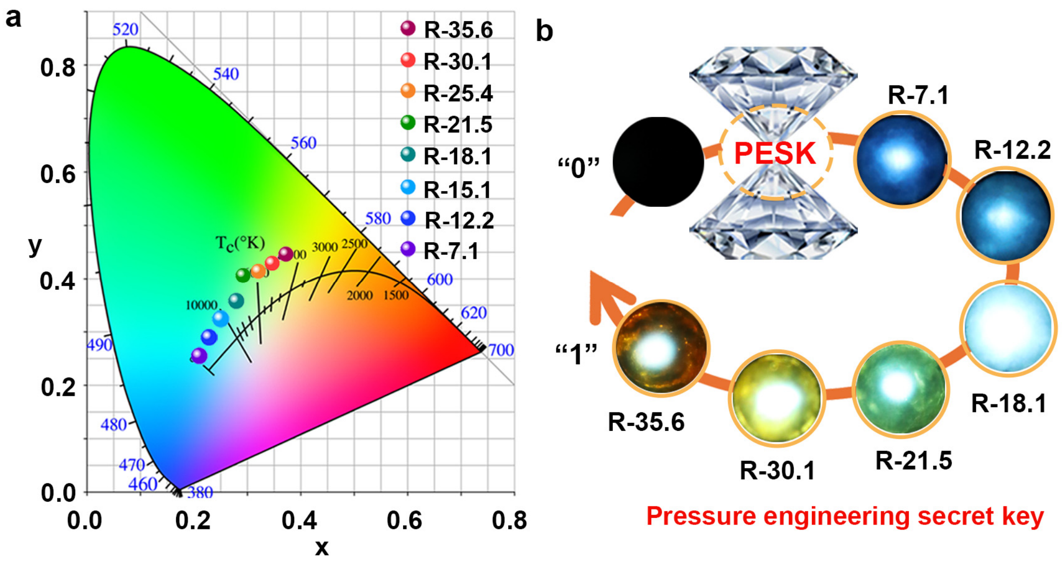

Supplementary Fig. 3 | Easily distinguished distinct colours of even for the naked eyes. a Commission Internationale de l'Eclairage (CIE) chromaticity diagram of $(4DMAP)_2ZnBr_4$ after pressure engineering. **b** Optical images of $(4DMAP)_2ZnBr_4$ at 1 atm under UV light illumination.

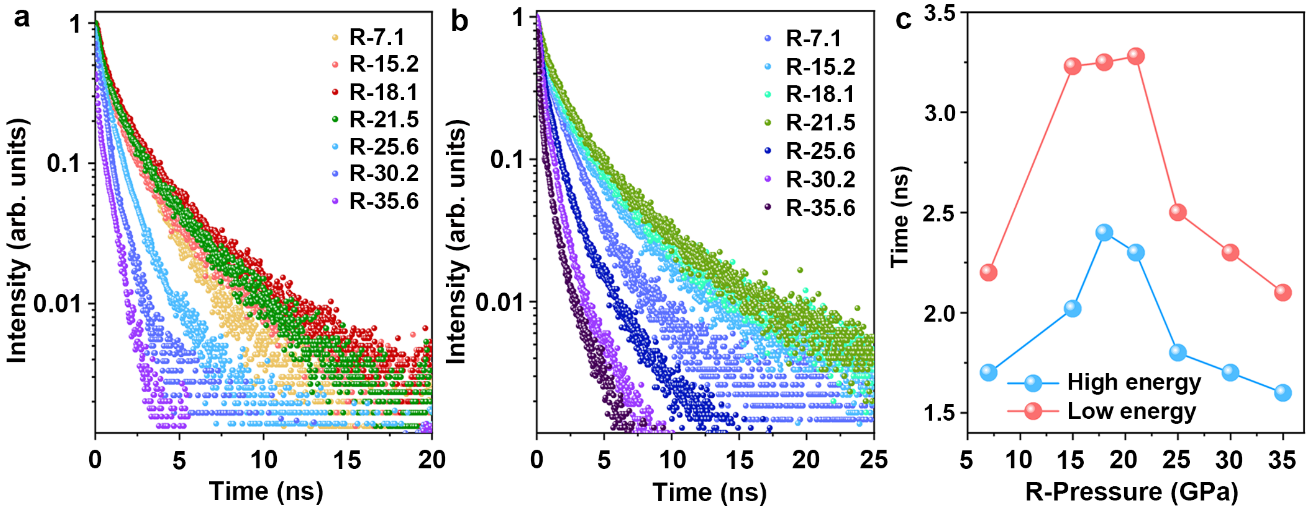

Supplementary Fig. 4 | PL decay curves after loading different pressures. a and **b** Changes in PL decay of high-energy and low-energy pinks of $(4DMAP)_2ZnBr_4$ after loading different pressures, respectively. **c** Lifetime vary in the samples R-7.1, …, 35.6 after loading different pressures.

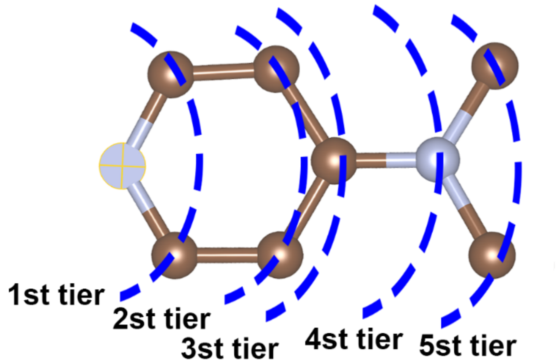

Supplementary Fig. 5 | Schematic diagram for calculating Steric Effect Index. The dotted line represents tiers of atoms in the 4DMAP^{+}.

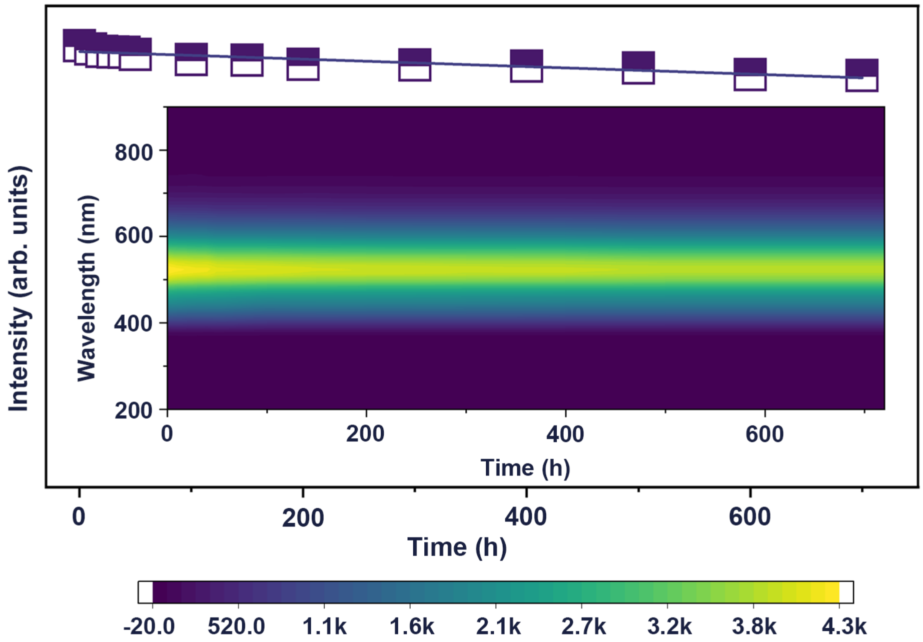

Supplementary Fig. 6 | Integrated PL intensity of $(4DMAP)_2ZnBr_4$ as a function of aging times Inset displays the corresponding PL intensity changes.

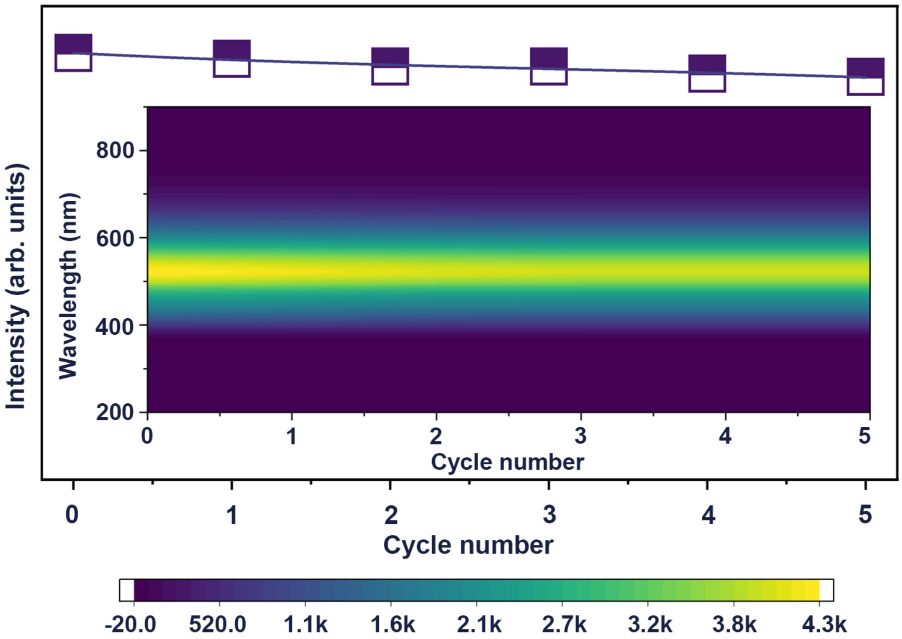

Supplementary Fig. 7 | Integrated PL intensity of quenched $(4DMAP)_2ZnBr_4$ as a function of cycle numbers by releasing from pressure of 21.5 GPa. Inset displays the corresponding PL intensity changes.

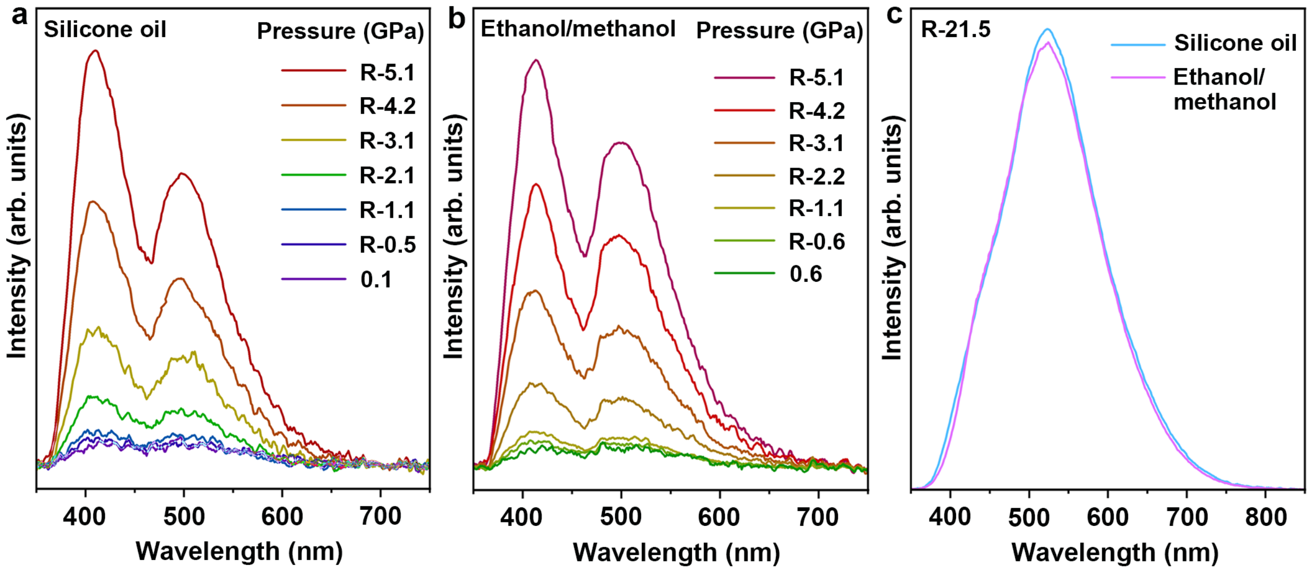

Supplementary Fig. 8 | Influence of the degree of hydrostaticity of the compression and threshold on the retainability of the optoelectronic response at ambient conditions. a and **b** Silicone oil and ethanol/methanol with a volume ratio of 4:1 used to be a pressure transmitting medium to investigate inflections of the degree of hydrostaticity of the compression. **c** Comparison of PL at R-21.5 GPa using silicone oil and ethanol/methanol as the pressure transmitting medium.

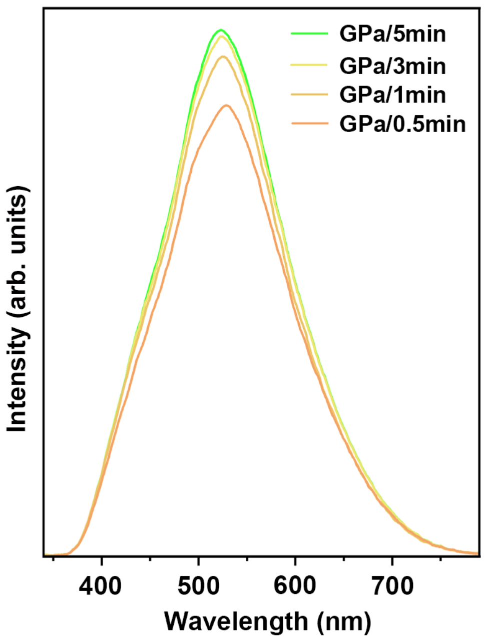

Supplementary Fig. 9 | Different kinetics in the pressure cycle R-21.5 GPa in response to the PL changes, including 1 GPa/5min, 1 GPa/3min, 1 GPa/1min, 1 GPa/0.5min.

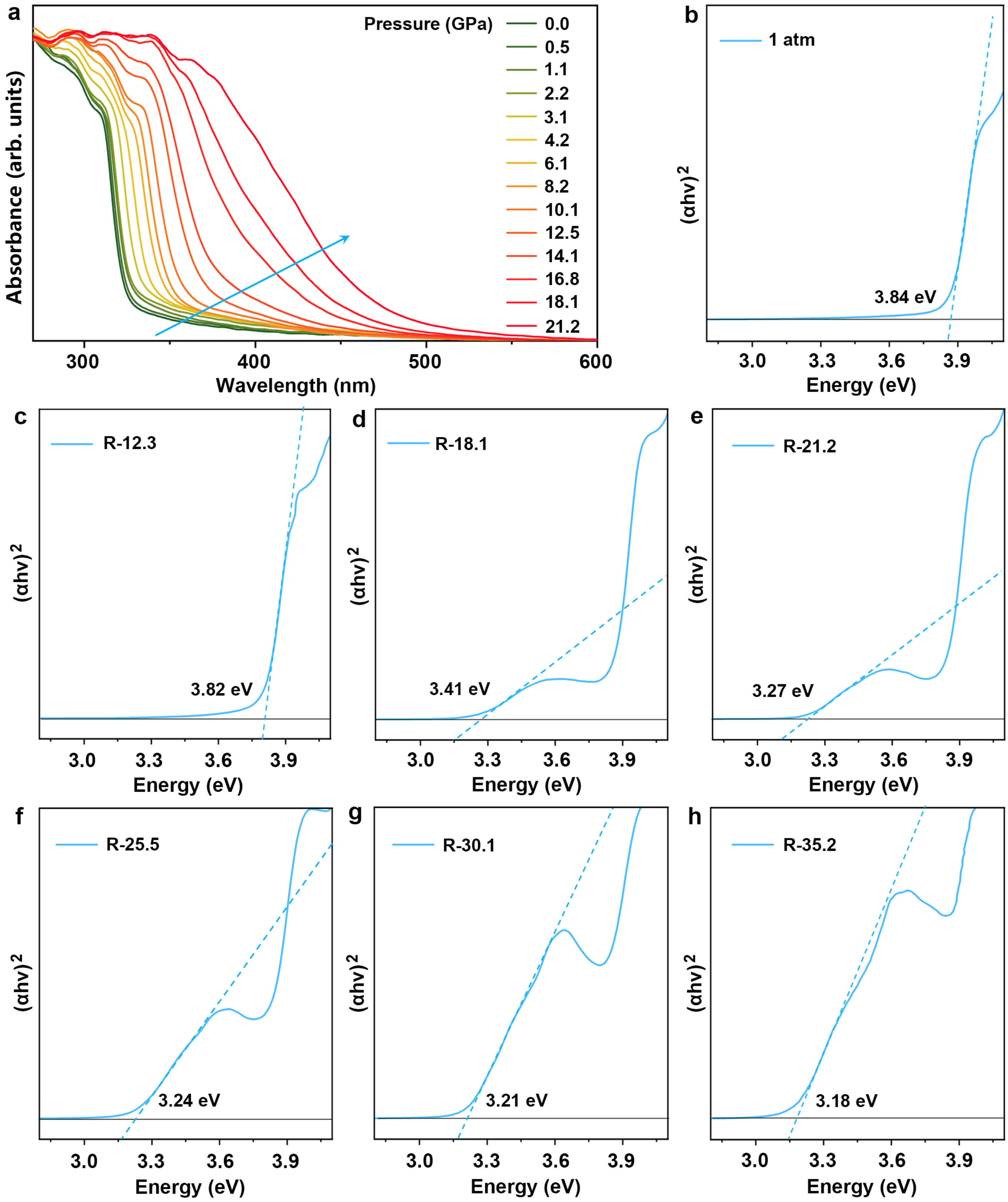

Supplementary Fig. 10 | Characterization of optical absorption under high pressure and decompression for $(4DMAP)_2ZnBr_4$. a Evolution process of absorption spectra under high pressure. **b-h** Comparison of absorption spectra between 1 atm and decompression from different pressures.

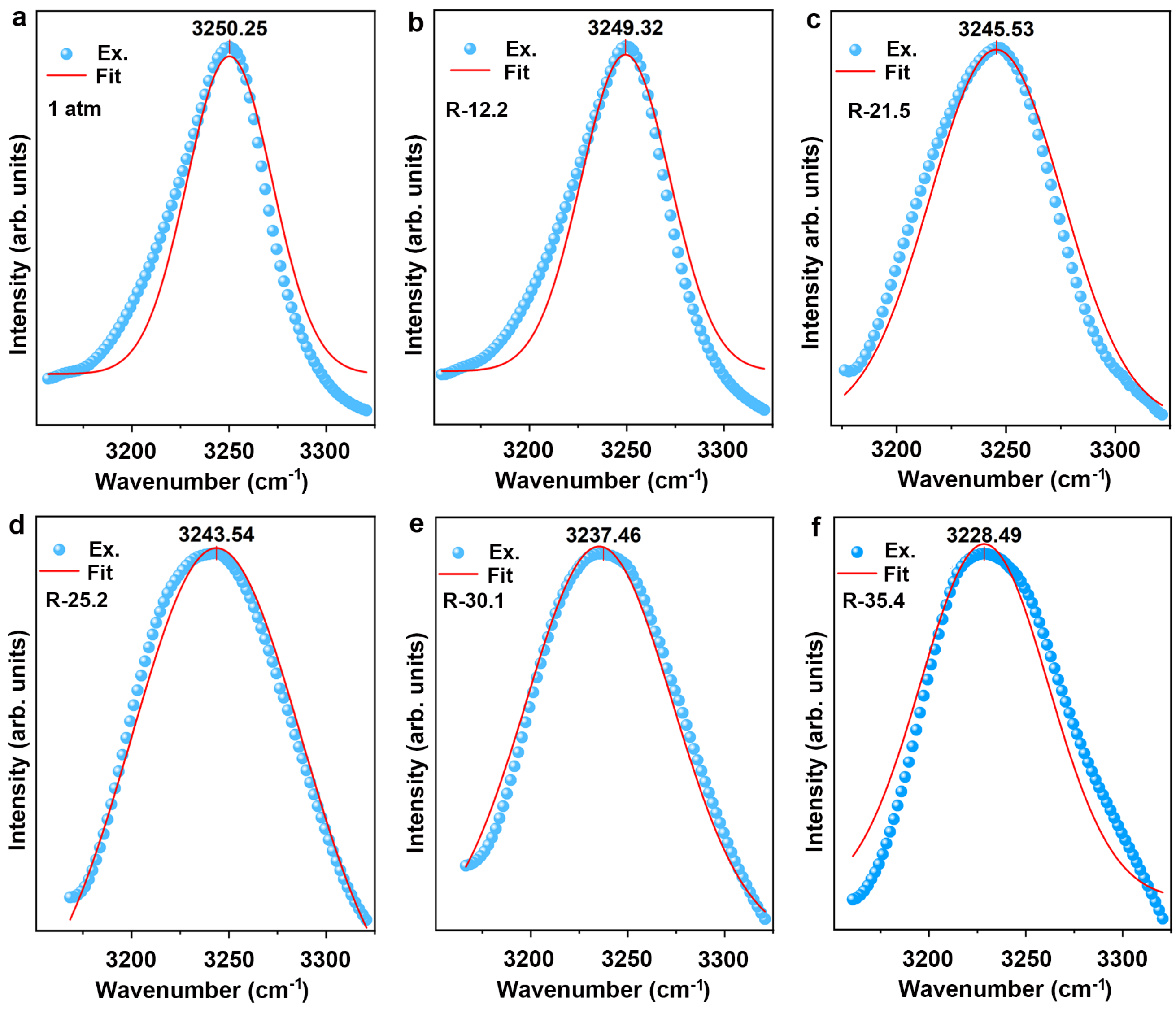

Supplementary Fig. 11 | Pink centers of IR vibrational spectrum. a-f Fitting procedure to extract the values for the peak centers using Gaussian after decompression from different pressures.

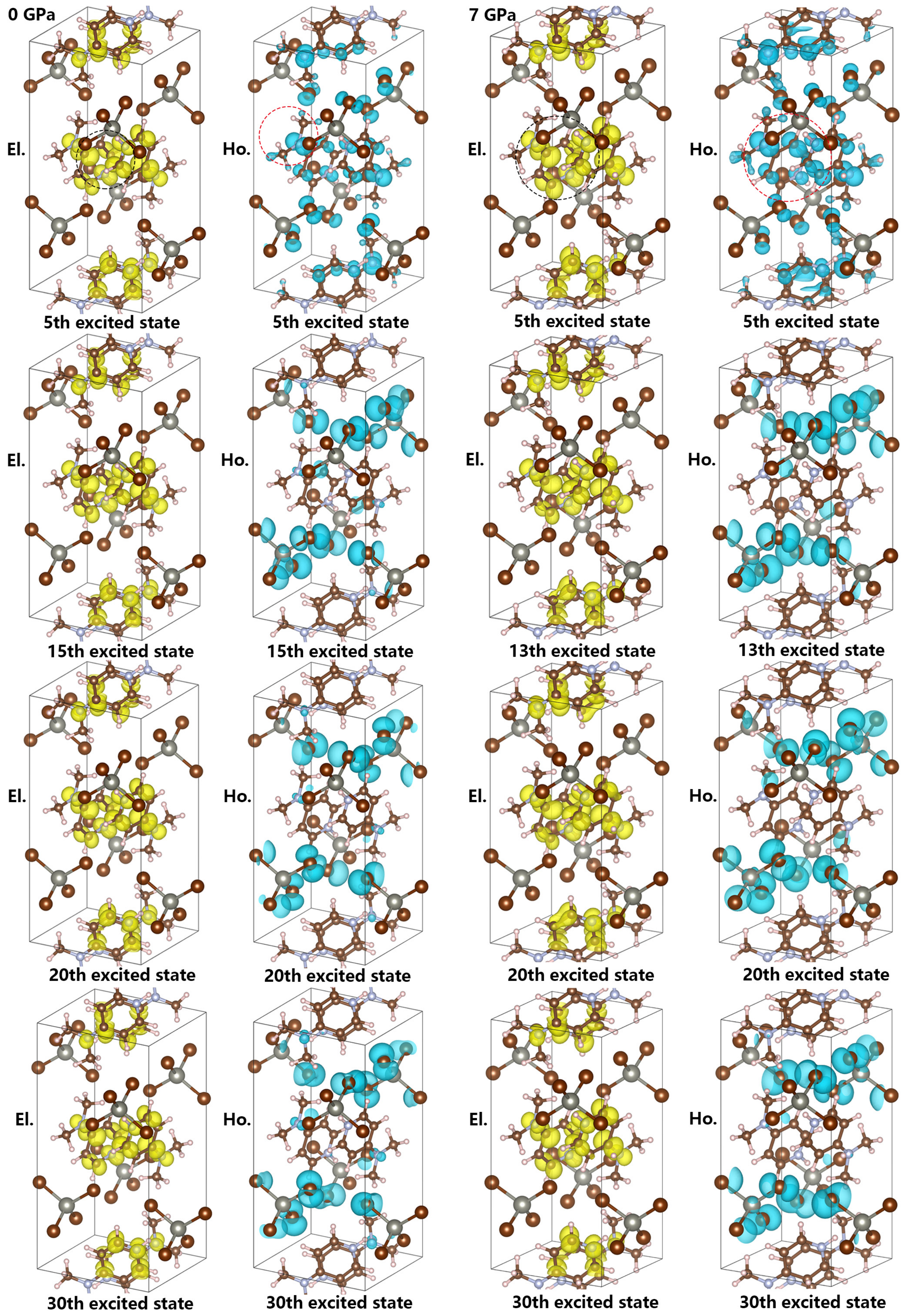
0 GPa
7 GPa
El.
Ho.
El.
Ho.
5th excited state
5th excited state
5th excited state
5th excited state
El.
Ho.
El.
Ho.
15th excited state
15th excited state
13th excited state
13th excited state
El.
Ho.
El.
Ho.
20th excited state
20th excited state
20th excited state
20th excited state
El.
Ho.
El.
Ho.
30th excited state
30th excited state
30th excited state
30th excited state

Supplementary Fig. 12 | Calculated wave functions of different excited states. a-p Hole and electron of 5th, 13th,15th, 20th 30th excited states at 1 atm and at 7 GPa, respectively. Here, El.: electron; Ho.: Hole.

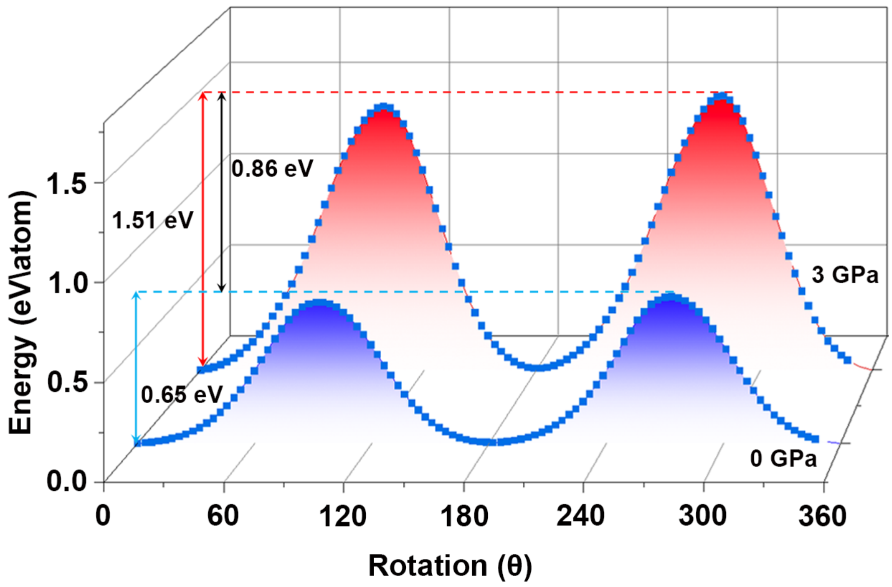

Supplementary Fig. 13 | Single-point energy for the rotation of $DMAP^{+}$ rings in $(4DMAP)_2ZnBr_4$. Single-point energy at 1 atm (blue) and at 3 GPa (red), respectively.

Supplementary Table I | Calculated single-point energy for the rotation of $4DMAP^+$ rings in $(4DMAP)_2ZnBr_4$ using DFTB+ calculations for 0 GPa and 3 GPa.

Rotation angle (°)		0 GPa (kcal/mol)		3 GPa (kcal/mol)
0		-72959.18		-72885.19
10		-72956.08		-72876.58
20		-72947.26		-72852.89
30		-72931.68		-72815.75
40		-72903.61		-72757.91
50		-72860.54		-72688.01
60		-72803.84		-72590.24
70		-72735.61		-72474.83
80		-72647.79		-72370.78
90		-72652.96		-72306.45
100		-72658.26		-72331.54
110		-72693.38		-72282.08
120		-72750.44		-72331.54
130		-72817.09		-72446.71
140		-72873.56		-72573.01
150		-72914.96		-72693.47
160		-72940.99		-72786.22
170		-72954.17		-72843.28
180		-72958.35		-72874.69
190		-72955.61		-72884.64
200		-72946.98		-72875.98
210		-72931.46		-72853.13
220		-72903.51		-72756.54
230		-72862.82		-72683.23
240		-72805.91		-72579.74
250		-72733.28		-72463.01
260		-72666.31		-72366.42
270		-72640.49		-72291.58
280		-72647.98		-72254.35
290		-72686.41		-72309.16
300		-72747.12		-72430.31
310		-72815.73		-72563.34
320		-72873.91		-72688.62
330		-72915.66		-72783.83
340		-72941.78		-72842.51
350		-72955.11		-72874.58
360		-72959.18		-72885.19

The rotational barriers were determined using the Density Functional based Tight Binding method (DFTB+) within VASP software.[1] The angle between $[ZnBr_4]^{2-}$ and $4DMAP^+$ shifted by 10 degrees for consecutive single-point energy calculations at each angle change (refer to Supplementary Figs. 13,14 and Supplementary Tables I, II).

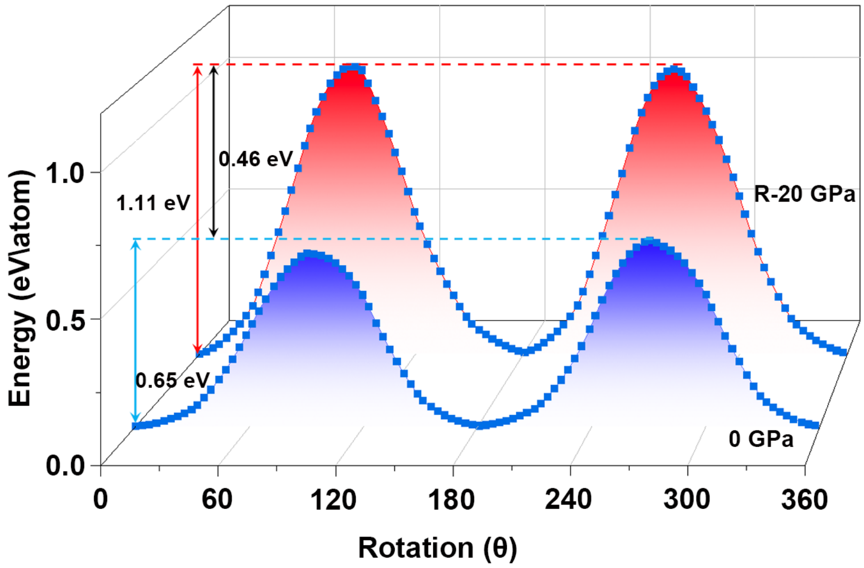

Supplementary Fig. 14 | Calculated Single-point energy for the rotation of $DMAP^+$ rings in $(4DMAP)_2ZnBr_4$. Single-point energy at 1 atm (blue) and at R-20 GPa decompressed from 20 GPa (red), respectively.

Supplementary Table II | Calculated single-point energy for the rotation of 4DMAP$^+$ rings in $(4DMAP)_2ZnBr_4$ using DFTB+ calculations for 0 GPa and R-20 GPa.

Rotation angle (°)		0 GPa (kcal/mol)		R-20 (kcal/mol)
0		-72959.18		-72929.23
10		-72956.08		-72920.14
20		-72947.26		-72893.11
30		-72931.68		-72843.81
40		-72903.61		-72764.74
50		-72860.54		-72661.08
60		-72803.84		-72554.83
70		-72735.61		-72464.27
80		-72647.79		-72420.08
90		-72652.96		-72444.17
100		-72658.26		-72502.71
110		-72693.38		-72593.36
120		-72750.44		-72692.69
130		-72817.09		-72771.01
140		-72873.56		-72828.01
150		-72914.96		-72874.16
160		-72940.99		-72901.51
170		-72954.17		-72918.83
180		-72958.35		-72926.82
190		-72955.61		-72919.64
200		-72946.98		-72893.53
210		-72931.46		-72846.37
220		-72903.51		-72771.62
230		-72862.82		-72667.93
240		-72805.91		-72565.26
250		-72733.28		-72473.85
260		-72666.31		-72427.84
270		-72640.49		-72447.59
280		-72647.98		-72502.82
290		-72686.41		-72506.54
300		-72747.12		-72694.55
310		-72815.73		-72773.45
320		-72873.91		-72828.15
330		-72915.66		-72874.29
340		-72941.78		-72904.44
350		-72955.11		-72921.95
360		-72959.18		-72928.94

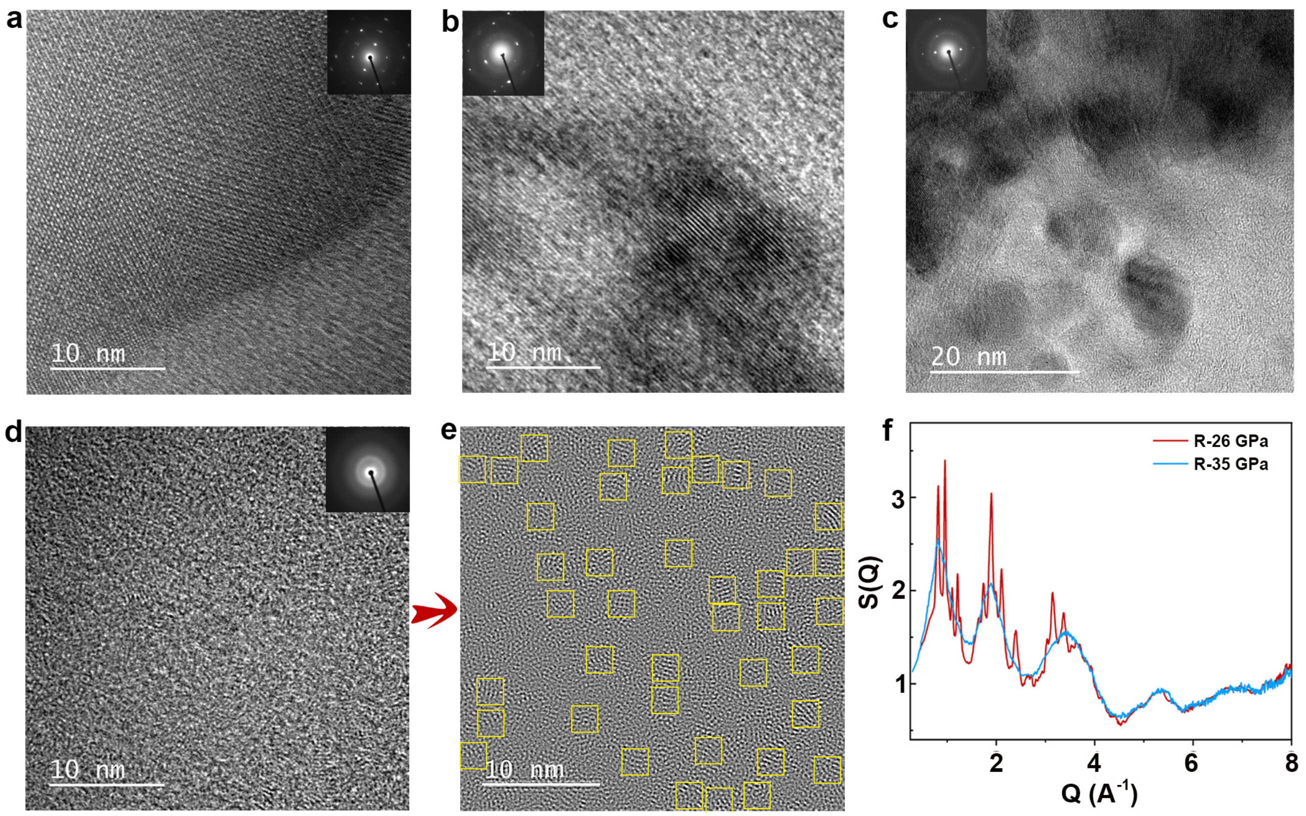

Supplementary Fig. 15 | Microstructure characterization. a-d HRTEM of products recovered from 1 atm, 18, 26 and 35 GPa, respectively. Inset represents selected area electron diffraction (SAED). **e** Inverse FFT image of the area in c. **f** Structure factor S(Q) of products recovered from 26 and 35 GPa.

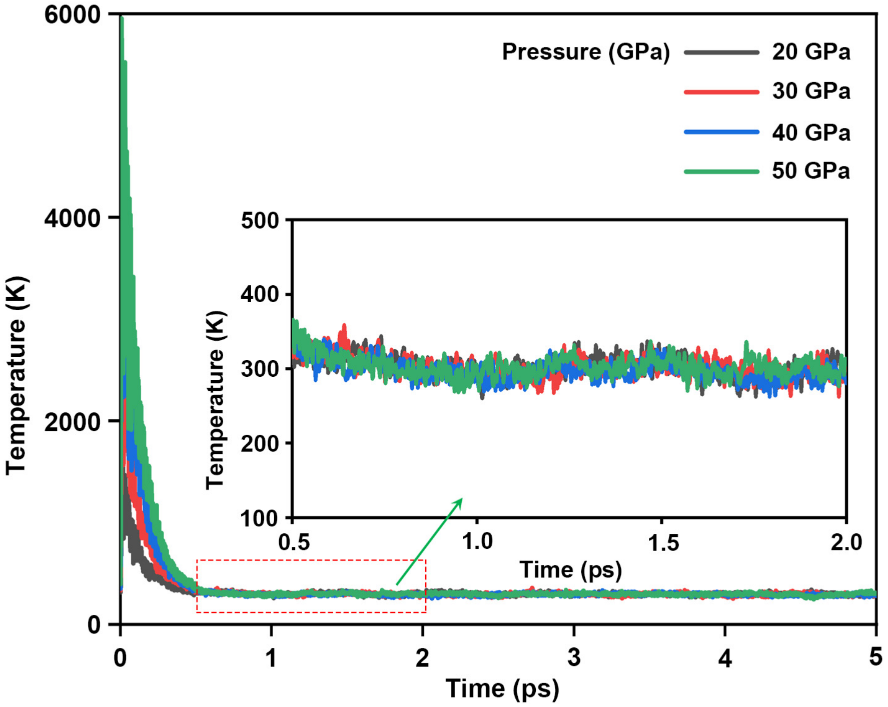

Supplementary Fig. 16 | Molecular dynamic simulation during different pressures. Relationship between temperature and time under different pressures during the molecular dynamic simulation process.

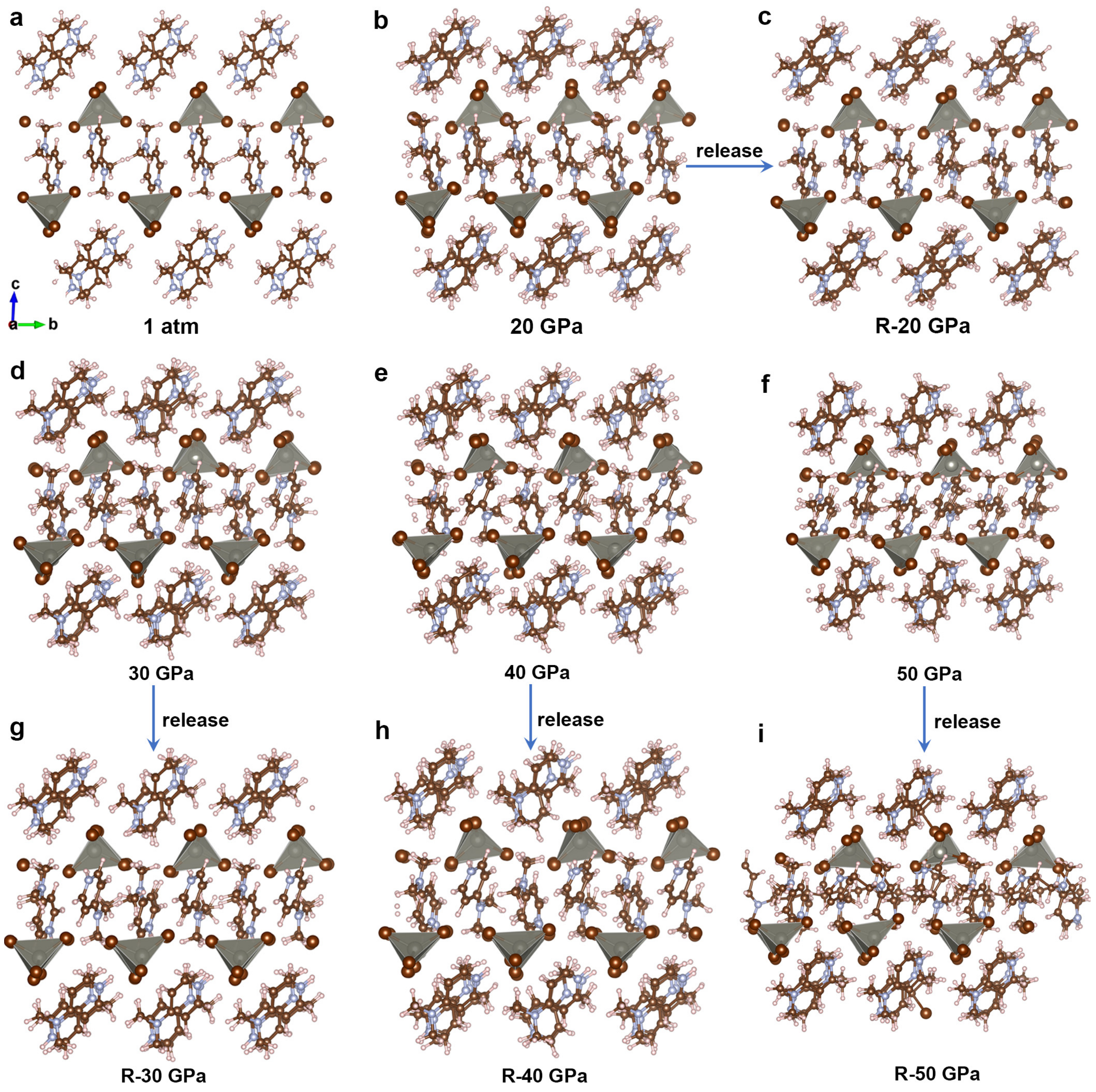

Supplementary Fig. 17 | Structural characteristics. Comparison of structures under ambient conditions and decompression from different pressures to ambient conditions.

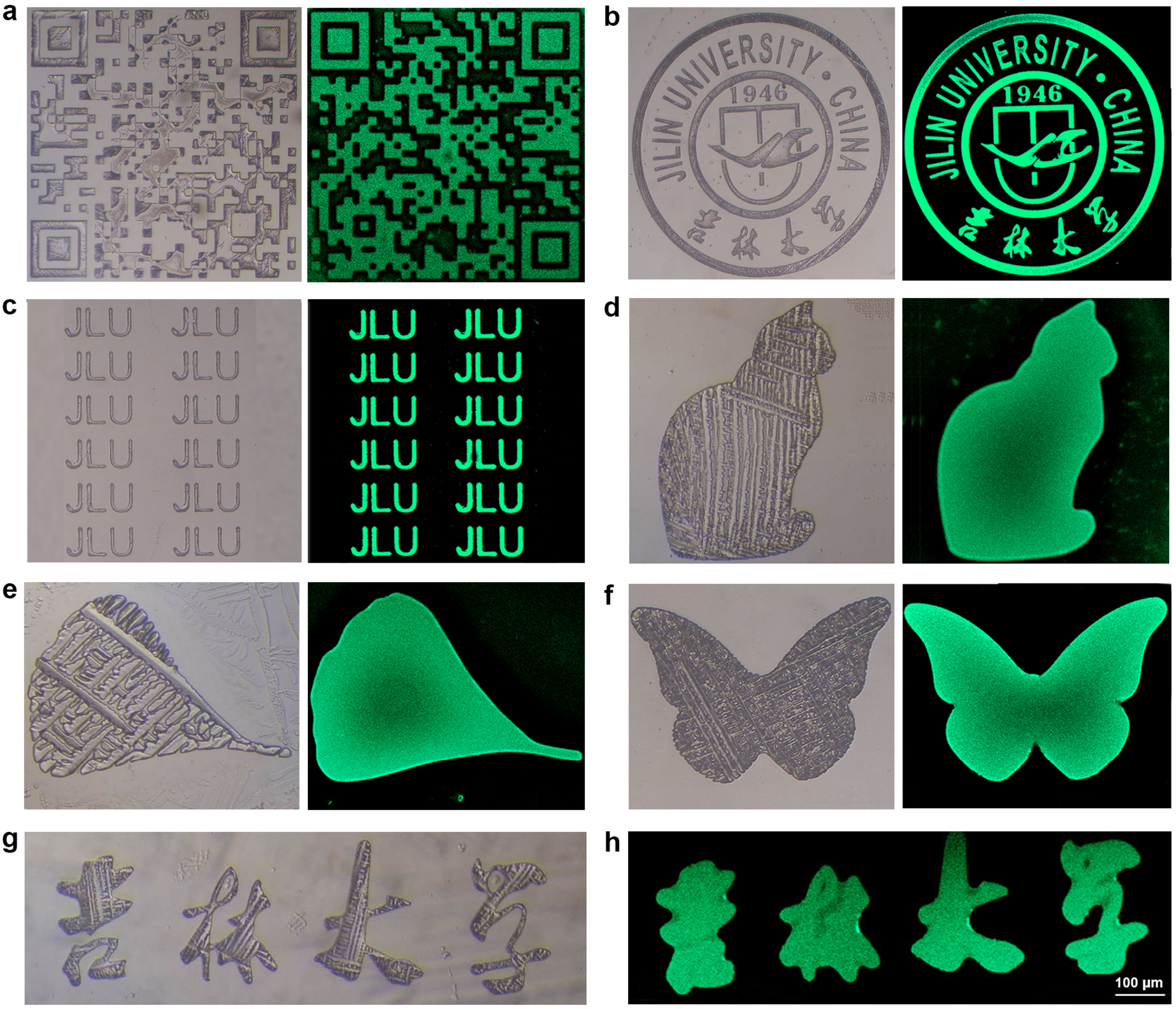

Supplementary Fig. 18 | Comparison of micro-nano patterns of $(4DMAP)_2ZnBr_4$ between 1 atm (left) and decompression (right). a-h Micro-nano patterns including non-emissive and emissive QR codes, school logos, school abbreviations, cats, ginkgo leaves, and butterflies, respectively (scale bar: 100 μm).

Supplementary references

1 van der Heide, T., Aradi, B., Hourahine, B., Frauenheim, T. & Niehaus, T. A. Hybrid functionals for periodic systems in the density functional tight-binding method. *Phys. Rev. Mater.* **7**, 063802 (2023).
